# The Crosstalk between Vitamin D and Pediatric Digestive Disorders

**DOI:** 10.3390/diagnostics12102328

**Published:** 2022-09-27

**Authors:** Cristina Oana Mărginean, Lorena Elena Meliț, Reka Borka Balas, Anca Meda Văsieșiu, Tudor Fleșeriu

**Affiliations:** 1 Department of Pediatrics I, “George Emil Palade” University of Medicine, Pharmacy, Science, and Technology of Târgu Mureș, Gheorghe Marinescu Street No 38, 540136 Târgu Mureș, Romania; 2 Department of Infectious Disease, “George Emil Palade” University of Medicine, Pharmacy, Science, and Technology of Târgu Mureș, Gheorghe Marinescu Street No 38, 540136 Târgu Mureș, Romania; 3 Department of Infectious Disease, County Clinical Hospital Târgu Mureș, Gheorghe Doja Street No 89, 540394 Târgu Mureș, Romania

**Keywords:** vitamin D, digestive disorders, children

## Abstract

Vitamin D is a cyclopentane polyhydrophenanthrene compound involved mainly in bone health and calcium metabolism but also autophagy, modulation of the gut microbiota, cell proliferation, immune functions and intestinal barrier integrity. The sources of vitamin D include sunlight, diet and vitamin D supplements. Vitamin D3, the most effective vitamin D isoform is produced in the human epidermis as a result of sunlight exposure. Vitamin D undergoes two hydroxylation reactions in the liver and kidney to reach its active form, 1,25-dihydroxyvitamin D. Recent studies highlighted a complex spectrum of roles regarding the wellbeing of the gastrointestinal tract. Based on its antimicrobial effect, it was recently indicated that vitamin D supplementation in addition to standard eradication therapy might enhance *H. pylori* eradication rates. Moreover, it was suggested that low levels of vitamin D might also be involved in the acquisition of *H. pylori* infection. In terms of celiac disease, the negative effects of vitamin D deficiency might begin even during intrauterine life in the setting of maternal deficiency. Moreover, vitamin D is strongly related to the integrity of the gut barrier, which represents the core of the pathophysiology of celiac disease onset, in addition to being correlated with the histological findings of disease severity. The relationship between vitamin D and cystic fibrosis is supported by the involvement of this micronutrient in preserving lung function by clearing airway inflammation and preventing pathogen airway colonization. Moreover, this micronutrient might exert anticatabolic effects in CF patients. Inflammatory bowel disease patients also experience major benefits if they have a sufficient level of circulating vitamin D, proving its involvement in both induction and remission in these patients. The findings regarding the relationship between vitamin D, food allergies, diarrhea and constipation remain controversial, but vitamin D levels should be monitored in these patients in order to avoid hypo- and hypervitaminosis. Further studies are required to fill the remaining gaps in term of the complex impact of vitamin D on gastrointestinal homeostasis.

## 1. Introduction

**Figure 1:**
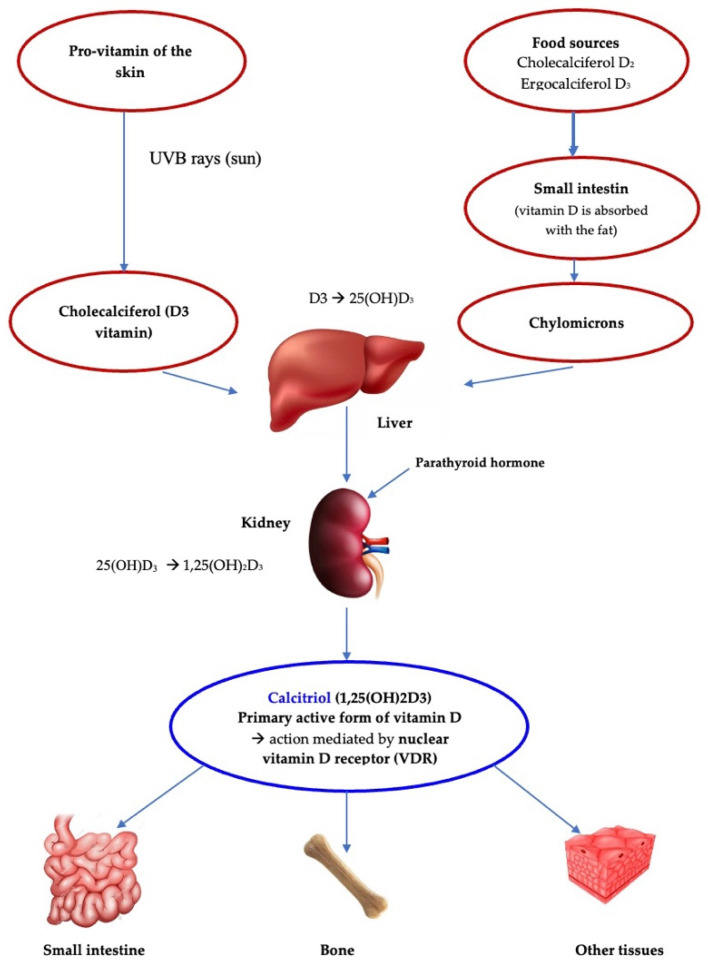
Schematic representation of vitamin D sources and metabolism.

Vitamin D belongs to the group of fat-soluble vitamins, which can be found in two compounds: cholecalciferol (vitamin D2) and ergocalciferol (vitamin D3). This vitamin can be obtained from a pro-vitamin of the skin in the presence of ultraviolet B rays from the sun by conversion in cholecalciferol or from alimentation by consumption of fish, mushrooms, dairy products or some supplements [1]. Vitamin D undergoes two hydroxylation reactions, first at the level of the liver, where it is transformed into 25(OH)D (25-hydroxyvitamin D), which is also the main circulation form, and then at the level of the kidneys, where it is further converted into 25-(OH)2D (1,25-dihydroxyvitamin D), also known as calcitriol, the active form of the vitamin (Figure 1). The functions of calcitriol in the body are mediated by the nuclear vitamin D receptor (VDR), expressed in various tissues such as the skin, adipocytes, small intestine, colon, parathyroid, etc. VDR and the retinoic acid receptor (RXR) form a heterodimer, which, at the level of the nucleus, binds to the vitamin D response element (VDRE), which involved in nuclear transcription regulation [1,2,3]. VDRE is found in multiple genes, explaining certain vitamin D-associated activities, such as autophagy [4], modulation of the gut microbiota [4,5,6], cell proliferation [7], immune functions and the intestinal barrier role [8,9]. Nevertheless, regulation of bone health and governance of calcium homeostasis remain the most important and well-documented roles of vitamin D [2,3,5]. Thus, vitamin D is essential for bone mineralization and bone mass development (Figure 1). 

The most common circulating form of vitamin D is 25(OH)D, which is also the best indicator for monitoring vitamin D status. In terms of vitamin D status, most studies have been performed on adults and define severe vitamin D deficiency as 25(OH)D levels < 10 ng/mL, resulting in an increased risk of rickets even when calcium intake is adequate, whereas in the presence of improper calcium intake, a level of 10–15 ng/mL vitamin D also leads to a high chance of developing rickets [10,11,12]. In adults, a vitamin D level of at least 20 ng/mL prevents the occurrence of rickets in up to 97.5% of the population, whereas levels ≥ 30 ng/mL have been proven sufficient to assure bone health [13,14]. In the pediatric population, the definition of an accurate cutoff level for vitamin D is difficult to establish. Despite the positive association between vitamin D levels, bone mineral density and bone mineral content revealed by pediatric studies, no specific threshold has been identified [15]. Moreover, it has been proven that calcium absorption in children might display an age-related compensatory mechanism that does not rely on vitamin D levels [16,17,18]. Similarly, a Chinese study performed on pediatric subjects aged between 0 and 7 years underlined that in the setting of 25(OH)D levels >30 ng/mL, the prevalence of decreased tibial bone mineral density plateaued [19]. Nevertheless, vitamin D deficiency and hypovitaminosis D were proven to be extremely common, irrespective of age. The National Health and Nutrition Examination in the US reported a prevalence of vitamin D deficiency of between 9–18% and 51–61% for hypovitaminosis D [20,21]. In Europe, a meta-analysis that involved 14,971 pediatric subjects identified a varying prevalence of vitamin D deficiency depending on age: 4–7% between 1 and 6 years, 1–8% between 7 and 14 years and 12–40% between 15 and 18 years, revealing that the prevalence increases with age [22]. Moreover, the authors found a higher prevalence in subjects living in relatively mid-latitude countries and non-white individuals. 

The skin is the most important organ, involved in up to 90% of vitamin D synthesis as a result of sun ultraviolet B radiation exposure, which is influenced by skin pigmentation, altitude, latitude, daily timing of sun exposure, seasonality, atmospheric pollution, type of clothing, percentage of skin area exposed and sunscreen use [23]. Based on their higher body surface-area-to-volume ratio and their augmented capacity to produce vitamin D, children clearly require less sunlight exposure compared to adults in order to produce sufficient amounts of vitamin D for proper bone mineralization and development [24]. Most foods, including breast milk, contain only low amounts of vitamin D and are considered insufficient as sources of vitamin D [25,26]. Thus, experts worldwide agree that vitamin D should be supplemented independent of the dietary habits. In the United Kingdom, the Scientific Advisory Committee on Nutrition recommends a safe vitamin D intake of 340–400 IU/day in infants, 400 IU/day in children aged between 1 and 4 years and 400 IU/day for the population aged 4 years and older [27]. The European Union recommendations differ, suggesting that an intake of 400 IU/day in infants between 7 and 11 months and of 600 IU/day in pediatric subjects aged 1–17 years might be sufficient to prevent further complications due to vitamin D deficiency [28]. Nevertheless, according to both the European Society for Pediatric Gastroenterology, Hepatology and Nutrition [29] and the European Academy of Pediatrics [30], the tolerable upper intake levels of vitamin D are 1000 IU/day in infants, 2000 IU/day for children aged between 1 and 10 years and 4000 IU/day for those aged between 11 and 17 years [31]. 

Besides the historical skeletal functions of vitamin D, in recent years it has been emphasized that vitamin D is involved, in a direct or indirect manner, in the regulation of up to 1250 genes, implying a wide spectrum of extraskeletal roles [23]. 

Thus, *the aim* of this review was to assess the role of vitamin D in the development of gastrointestinal disorders. 

## 2. Vitamin D and Gastrointestinal Disorders

Based on the wide spectrum of immune-modulatory properties of vitamin D, its involvement in the development of gastrointestinal disorders is not surprising. Vitamin D deficiency has been proven to result in severe dysfunctions of the intestinal barrier [32], mucosal damage [33] and increased susceptibility to infectious agents, thus altering the development and maintenance of gut homeostasis [34]. On the contrary, adequate levels of vitamin D have been associated with the integrity of junction complexes, therefore protecting the intestine from injury [35,36]. 

### 2.1. Vitamin D, Gastritis and Gastroesophageal Reflux

The role of vitamin D in modulating the host’s response to pathogens and foreign antigens, along with the properties of vitamin D receptors as regulators of the immune system, attracted considerable attention in the research community during the last century [37]. Several hypotheses were proposed as potential mechanisms to explain the involvement of vitamin D in host defense against infection, such as the ability of immune cells to produce cytochrome P450 family 27 subfamily B member 1 and to convert 25(OH)D into 1,25(OH)2D; the expression of vitamin D receptor by the majority of immune cells; the strong relationship between 1,25(OH)2D production in immune cells and subsequent synthesis of antibacterial products, such as β-defensin and cathelicidin; and the strong evidence that vitamin D deficiency augments the burden of infectious diseases worldwide [38]. Moreover, epidemiologic studies revealed that the altered status of vitamin D is closely involved in mitigating susceptibility to various pathogens [39]. The wide spectrum of vitamin-D-related functions in terms of the immune system includes stimulation of macrophages and activated B and T cells, as well as maturation of dendritic cells; production of neutral antibacterial peptides and proteins, as well as reactive oxygen species; and the expression of inducible nitric oxide synthase [40]. Moreover, children with rickets were found to be at increased risk of developing infections, especially those involving the respiratory tract [41] (Table 1). 

A recent study performed on subjects with *Helicobacter Pylori* (*H. pylori*) infection proved higher eradication rates if vitamin D supplements were combined with clarithromycin-based triple therapy [42], suggesting that vitamin D might also exert a protective antimicrobial effect against *H. pylori* infection. These findings were obtained in a study by Yildirim et al., who reported significantly lower eradication rates in deficient individuals [43]. Moreover, another study that included Italian subjects highlighted that patients with *H. pylori*-positive gastritis had lower serum vitamin D concentrations compared to uninfected individuals [44]. Similarly, Guo et al. [45] reported that vitamin D plays a major role in gastric mucosa homeostasis, exerting an antimicrobial effect against *H. pylori* based on its implications in sustaining host defense mechanisms. Thus, in patients with vitamin D deficiency, the infected macrophages no longer produce sufficient amounts of 1,25-(OH)D2, altering the subsequent synthesis of antimicrobial peptides and proteins and eventually disabling their ability to suppress and kill *H. pylori* strains [46]. Another potential explanation for the positive association between vitamin D levels and *H. pylori* eradication was suggested by an in vitro study that proved that the vitamin D3 decomposition product has a selective antibacterial effect against *H. pylori* [47]. In addition, vitamin D deficiency is associated with the augmentation of T-cell aggression in patients with chronic *H. pylori* infection, worsening gastric mucosal injury [44]. A recently reported case in an 18-month-old child underlined that hypovitaminosis D might result in an exacerbation of *H. pylori* gastritis, despite the young age of the patient [48] (Table 1). 

As previously mentioned, gut epithelial VDR signaling seems to play a crucial role in regulating mucosal inflammation; although vitamin D is not produced in the stomach, it alters immune regulatory responses through the presence of this receptor within the stomach [45,49]. Studies proved that VDR mRNA expression levels were significantly increased in patients with *H. pylori* infection and were positively correlated with the activity scores of chronic inflammation [45]. Animal studies also revealed out a strong association between VDR and *H. pylori* infection, with VDR knockdown mice experiencing an increased susceptibility to this infection [50] (Table 1). 

Chronic atrophic autoimmune gastritis is another pathology worth mentioning in the discussion of vitamin D. In addition to vitamin B12 deficiency and iron malabsorption, which are the most common disturbances in patients with this condition, other deficiencies were also recently described in these patients, including vitamin D deficiency [51] (Table 1). 

The relationship between vitamin D and gastroesophageal reflux is less studied than the effect of vitamin D on gastritis. Thus, a recent study with the aim of assessing vitamin D intake and vitamin D levels in children with gastroesophageal reflux disease reported that children with this pathology had a normal level of vitamin D, despite a level of vitamin D intake below the daily recommended intake [52]. 

Undoubtedly, there is a strong relationship between vitamin D levels in children and *H. pylori*-positive gastritis, and vitamin D most likely also impacts other types of gastritis and even gastroesophageal reflux disease. However, further studies are required to define the precise role of this vitamin in the pathophysiology of these disorders. 

### 2.2. Vitamin D and Celiac Disease

It is well-documented that celiac disease (CD) is an autoimmune disorder of the gastrointestinal tract triggered by an immune response to gluten-containing grains in individuals carrying HLA class II molecules HLA-DQ2 and HLA-DQ-8 [53]. The prevalence of CD in the US and most of European countries is approximately 1% [54]. Early-life vitamin D deficiency might be linked to the development of CD < 15 years of age [55]. The main mechanism implicated in childhood-onset CD involves the dysregulation of the intestinal response in deficient subjects, resulting in a disruption of the epithelial barrier, further increasing gluten permeability [56]. Moreover, the negative effects of vitamin D deficiency might begin during the intrauterine life, several studies have highlighted that low vitamin D concentrations in pregnant women may negatively affect fetal development, increasing the offspring’s susceptibility to developing both infections [57,58] and autoimmune disorders [59,60] (Table 1). Nevertheless, the results on this topic remain controversial, as although the authors of some studies have hypothesized the role of maternal vitamin D deficiency in the onset of CD [61,62], a recent study that tested this hypothesis revealed no association between gestational or early-life vitamin D levels and the development of childhood CD [63] (Table 1). 

The core of the complex pathophysiology of CD is represented by the intestinal barrier. The gut barrier is a complex structure meant to prevent harmful agents from passing though the gut mucosa and reaching the lamina propria [64]. The proper functioning of this barrier is closely related to epithelial layer integrity, intestinal microbiota homeostasis and gut-associated lymphoid tissue health [65,66,67]. Although the main trigger of all pathological events in CD is gluten, it seems that early disruption of the intestinal barrier in susceptible individuals and subsequent increased permeability could also contribute to the onset of immune responses triggered by gluten [65,66]. Aside from the positive effect of vitamin D on lymphocyte T and dendritic cells, this vitamin in also involved in the regulation of gut barrier integrity based on its close interaction with tight junctions, enabling the suppression of the zonulin release signaling pathway to upregulate tight junction protein expression and to consequently suppress the increase in gut mucosa permeability [64,66]. Moreover, vitamin D was reported to be involved in regulating inflammatory cytokines, such as tumor necrosis factor α [68], adding to its crucial role regarding gut barrier integrity maintenance (Table 1). 

Children with CD are commonly reported to have low vitamin D levels, and it is difficult to establish whether this deficiency is a result of their dietary restrictions or whether vitamin D deficiency is involved in the onset of childhood CD. This phenomenon is most likely a vicious circle in which general trends of hypovitaminosis D during childhood contribute to the onset of CD, and its persistently decreased levels during the clinical course of CD augment disease severity. A recent study indicated that the decreased expression of VDR and epithelial barrier proteins claudin-2 and E-cadherin are positively correlated with histological findings of disease severity [69]. The authors pointed out that this decreased expression of VDR and epithelial barrier proteins is the result of vitamin D deficiency. Similar findings were reported by Zanchi et al., who found that 35% of biopsy-proven CD children included in their study experienced vitamin D deficiency [70]. On the contrary, Villanueva et al. failed to identify a positive association regarding vitamin D levels in healthy children and those with CD [71]. As previously stated, vitamin D deficiency is also common after the initiation of a gluten-free diet, most likely due to imbalances in calcium and vitamin D levels caused by this type of diet [72]. Although gluten-free diet was associated with considerable improvement in bone mineral density after only one year [73], the complete normalization of bone mineral density cannot always be achieved only by excluding dietary gluten [72]. Multiple controversies emerged regarding the supplementation of vitamin D in patients with CD based on the contradictory findings reported in the literature. The authors of a study that assessed children and teenagers with CD reported that a 2-year course of vitamin D (400 UI/day) and calcium (1 g/day) supplementation had a positive impact on bone mineral density [74]. On the contrary, another study proved that a gluten-free diet alone lasting for approximately 6 months was sufficient to resolve hypocalcemia and hyperparathyroidism, as well to normalize vitamin D levels [70]. Therefore, the choice to supplement vitamin D in combination with a gluten-free diet in patients with CD should be judiciously weighed (Table 1). 

It has been reported that at the time of diagnosis, low bone mineral density might be present in CD patients, regardless of symptomatic or asymptomatic status [75,76]. Thus, there is a global consensus that vitamin D levels should be assessed upon diagnosis of CD [23,77,78,79]. Nevertheless, a recent study reported no difference in mean 25-(OH)D in recently diagnosed CD patients compared to healthy controls [80]. 

Thus, the assessment of vitamin D levels is mandatory in children with CD upon diagnosis and even during the clinical course in order to avoid improper the use of vitamin D supplements. 

### 2.3. Vitamin D and Cystic Fibrosis (CF)

CF is an autosomal recessive disease and one of the most common causes of death related to this type of disorders among Caucasians in the US. The incidence of this disease is of 1 in 3200 live births, accounting for approximately 30,000 patients in the US and approximately 70,000 patients worldwide [81,82]. CF is caused by a mutation in the cystic fibrosis conductance regulator (CFTR) gene, resulting in an absent or dysfunctional CFTR protein-altering chloride transport through the apical epithelial membrane [83]. Several studies reported that the prevalence of vitamin D insufficiency is as high as 90% in the CF population [84,85]. A wide spectrum of factors was incriminated in the etiology of this deficiency, such as pancreatic insufficiency, reduction in body fat and vitamin D binding protein, reduced sunlight exposure and improper hepatic hydroxylation of vitamin D [86]. According to the Cystic Fibrosis Foundation, the serum 25(OH)D concentration should be determined annually, especially at the end of winter, and should be preserved > 30 ng/mL year round [87] (Table 1). 

Studies performed on both adults and children revealed that vitamin D deficiency (<10 ng/mL) in adults with advanced CF was positively correlated with low hip and spine bone mineral density as compared to CF patients with higher vitamin D levels, whereas in children, the concentration of the same vitamin was also positively correlated with femoral neck and lumbar spine Z scores [88,89]. Vitamin D may be also be involved in preserving lung function in patients with CF [90], as a positive correlation was identified between vitamin D status and lung function indicators, such as forced expiratory volume in 1 s and forced vital capacity [91]. Several mechanisms were suggested to explain the role of vitamin D in preserving lung function, such as the effect of this vitamin on decreasing airway inflammation, the impact on airway remodeling as a response to various injuries caused by CF and the ability to decrease airway bacterial colonization [92] (Table 1). 

Moreover, increased vitamin D levels were proven to be associated with both improved lung function and a reduction in the frequency of pulmonary exacerbation episodes [92]. In terms of lung exacerbations and infections, it was proven that locally produced 1,25(OH)2D enhances interleukin (IL)-37 airway concentrations, which further decrease airway colonization by certain pathogens, such as Pseudomonas aeruginosa and Bordetella bronchiseptica [93]. Vitamin D has the ability to downregulate proinflammatory cytokine synthesis in macrophages, resulting in a reduction in CF airway inflammation [94]. Another beneficial effect of vitamin D is reactive nitrogen and oxygen intermediate production, with a major effect on inducing autophagy to clear infections [95]. The authors of a randomized, controlled, double-blind placebo trial that included 30 CF adults hospitalized for a pulmonary exacerbation concluded that patients who were administered a one-time oral dose of 250,000 IU of vitamin D3 experienced more antibiotic therapy-free days and hospital-free days, as well as 1-year survival rate in comparison to those who received a placebo [96]. In addition, the authors pointed out that the high-dose vitamin D group presented with a significant decrease in inflammatory biomarkers, such as tumor necrosis factor α and IL-6. According to these findings, a high dose of vitamin D might represent a beneficial adjunctive therapy in CF patients with acute airway infections, which was also supported follow-up in a larger multicenter study performed by the CF Foundation [97] (Table 1). 

CF-related intestinal microbial dysbiosis has been well-documented to result from both bacterial overgrowth as a result of poor intestinal motility and alterations of bacterial populations due to commonly used antibiotic treatment [98]. Studies on animals revealed that vitamin D exerts strong anti-inflammatory effects in the intestine of CF patients, resulting in decreased synthesis of proinflammatory markers, such as nuclear factor kB and IL-8, as well as reduced eosinophilia levels within the duodenal mucosa and fewer apoptotic cells [99]. Multiple factors can alter the microbial population in CF patients, such as common pulmonary infections, malnutrition and repeated systemic or local antibiotic treatment [100], impairing microbial communities within both the lungs and intestine. A recent study that assessed the influence of vitamin D on the lung and intestinal microbiome of CF patients revealed that vitamin D treatment led to major changes in lung and intestinal microbiome compositions, enhancing the development of healthier intestinal and respiratory microbes [101] (Table 1).

Another emerging topic related to vitamin D and CF is represented by metabolomic studies. A study on CF adult patients hospitalized with lung exacerbations aimed to assess the role of vitamin D in biochemical and metabolomic studies by dividing patients into two groups: a vitamin D group, which received a single 250,000 IU bolus dose of vitamin D, and a placebo group [102]. The authors collected baseline serum samples prior to drug administration and 7 days later for metabolomic analysis and reported that the baseline metabolome of CF patients was mainly catabolic, implying increased markers of lipid and fatty acid metabolism and reduced levels of essential amino acids. The samples collected after drug administration revealed that the placebo group exhibited 15 more catabolic pathways involving carbohydrates, amino acids and lipid metabolism overall compared to the vitamin D group (Table 1).

Based on the aforementioned facts, prescribing daily or weekly supplements of vitamin D in CF patients seems a reasonable choice.

### 2.4. Vitamin D and Inflammatory Bowel Disease (IBD)

IBD comprises two major clinical entities, Chron’s disease (ChD) and ulcerative (UC), represents a chronic, relapsing–remitting systemic disorder with onset usually occurring during young adulthood and lasting throughout life [103]. Remission maintenance through medical therapy represents the cornerstone of management in IBD patients [104]. Vitamin D was proven to induce and maintain remission in IBD patients based on its anti-inflammatory and antibacterial properties, although it might also contribute to the repair of the intestinal mucosal barrier [105,106]. Moreover, vitamin D might also impact the incidence and progression of ChD and UC [107,108,109], as although controversial, it seems to be involved in the development of IBD and its severity according to several findings revealing common vitamin D deficiency in newly diagnosed IBD patients [104,110,111]. Similar findings were reported by Li et al., who found a significant association between vitamin D levels and IBD [103]. Moreover, previous studies highlighted that this micronutrient might also be involved in IBD-specific complications [112,113]. Studies with the aim of stratifying the impact of vitamin D on either UC or ChD remain controversial. Veit et al. [114] and El-Matary et al. [115] reported that vitamin D levels were higher in pediatric patients with UC as compared to those with ChD, whereas Li et al. found no significant differences between UC and ChD patients in terms of vitamin D levels [103] (Table 1).

The prevalence of vitamin D deficiency in IBD patients is significantly higher than that in other populations, independent of IBD type [116]. A systematic review and meta-analysis of 14 observational studies including 938 IBD patients underlined a prevalence of vitamin D deficiency of 38.1% in ChD patients and of 31.6% in those with UC [117]. Several studies assessed the predictors of vitamin D deficiency in IBD and reported IBD-related surgery, non-white ethnicity [118,119], African-American ethnicity and body mass index > 30 kg/m^2^ as the most common [120] (Table 1).

Vitamin D status also seems to be related to IBD activity. Worldwide studies [121,122,123,124,125,126,127] proved a significant association between vitamin D and disease activity, raising a ‘chicken or egg’ question, calling into doubt whether vitamin D levels are linked to intestinal inflammation or whether intestinal inflammation results in low vitamin D absorption. Nevertheless, more recent evidence suggests that serum vitamin D concentrations are inversely correlated with endoscopic and histologically proven inflammation, mucosal expression of proinflammatory cytokines and disease activity [128,129]. A reverse correlation was also highlighted between vitamin D levels and both fecal calprotectin and erythrocyte sedimentation rate [124]. Some studies have suggested that these findings were accurate only in ChD and not UC patients [126,130]. Other studies suggest that low or insufficient vitamin D levels are related to the increased need for hospitalization and surgery in patients with IBD compared to those with normal serum levels [131,132]. Another important aspect of disease activity in IBD patients is related to the gut microbiota, as it is well-documented that abnormal immune response to intestinal commensal bacteria is a specific feature of IBD, resulting in a less diverse and imbalanced microbial community [133,134,135]. Several promising effects of vitamin D on IBD gut microbiota modulation were recently reported by Battistini et al., suggesting its crucial positive role in modulating the composition of the gut microbial community [9] (Table 1).

In terms of vitamin D supplementation, IBD patients might require higher doses to achieve the recommended circulating level (>20 ng/mL) due to common nutrient malabsorption issues in these patients [13]. Nevertheless, supplementation has been reported to be beneficial, as it is associated with an improvement in inflammatory biomarkers, such as the erythrocyte sedimentation rate, C-reactive protein and suppression of the Th1 immune response, reducing the clinical disease activity index [136,137,138,139,140,141,142,143] (Table 1). Thus, the complex beneficial implications of vitamin D in the pathophysiology, outcome and disease activity of IBD should increase the awareness in clinical practice regarding the supplementation of this micronutrient in IBD patients. 

### 2.5. Vitamin D and Food Allergies

Recent evidence suggests that the prevalence of vitamin D insufficiency in the population increases in parallel with the prevalence of food allergies [144]. Moreover, the role of vitamin D insufficiency at the age of 12 moths in the development of food allergy was emphasized by a population-based study performed on infants in Melbourne [144]. Nevertheless, the precise mechanisms involved in this relationship remain unclear. It was proven that vitamin D receptor agonists influence Th1 and Th2 cell function by suppressing allergen-specific IgE synthesis, inhibiting the maturation of dendritic cells, inducing tolerogenic dendritic cells and eventually contributing to the induction of regulatory CD4^+^CD25^+^Foxp3^+^ T cells [145]. A potential mechanism described by Vassallo and Camargo implies the negative impact of vitamin D deficiency on the integrity of the gut barrier, resulting in increased permeability due to colonization by pathogenic microbial flora and subsequent inadequate immune system exposure to dietary allergens [146]. Another potential mechanism emphasizes the possibility of transcutaneous sensitization in children with vitamin D deficiency [147]. Thus, reduced antimicrobial factors at the skin level and the lack of effective tight junctions due to this deficiency might lead to inappropriate exposure and stimulation of the immune system, triggering the development of allergic sensitization, eczema and food allergy [147], in addition to worsening the evolution of atopic dermatitis [148]. The genetic aspect of the relationship between vitamin D deficiency and allergic disorders should not be neglected being, proving that certain individuals are more susceptible to developing food allergies, most likely due to differences in the genes involved in vitamin D metabolism and the response to vitamin D supplementation [149].

Recently, a worldwide increase in the prevalence of allergic diseases was reported [150,151]. Vitamin D deficiency was associated with the development of several allergic disorders, such as atopic dermatitis, asthma and food allergy [152]. A study conducted in Australia revealed that vitamin D levels < 50 nmo/L at 1 year of age were associated with an 11-fold increased risk of peanut allergy and a 4-fold increased risk of egg allergy [153]. Nevertheless, the question, ‘which came first: the chicken or the egg?’ also applies to this topic, as vitamin D deficiency was also reported in patients already diagnosed with food allergies, including cow’s milk allergy (CMA), due decreased intake [154]. Children with both IgE- and non-IgE-mediated food allergies were proven to be at increased risk of vitamin D deficiency [155,156,157]. On the contrary, it was recently suggested that increased levels of vitamin D might increase the likelihood of sensitization and food allergy [158]. Similarly, a recent review revealed no benefit of vitamin D supplementation with respect to primary allergy prevention [159]. A recent Japanese study concluded that the correction of vitamin D deficiency might have a positive impact on food allergy prognosis, emphasizing the domino effect between inadequate sunlight exposure, vitamin D deficiency, altered gut barrier integrity, impaired immune response and food allergies [160]. 

The contradictory findings reported regarding the role of vitamin D in the development of food allergies represent proof that further studies are required in order to clearly delineate the effect of vitamin D on patients with food allergies and the effect of food allergies on vitamin D status. However, vitamin D status should be closely monitored in children with food allergies in order to assure its appropriate level for the best outcome. 

#### Vitamin D and Cow’s Milk Allergy (CMA)

Taking into account that CMA is one of the most common food allergy in infants [161], we considered that it would be useful to briefly discuss certain aspects regarding the relationship between vitamin D and CMA. It is essential to underline that cow’s milk composition contributes to normal development and growth, especially during early childhood; its micro- and macronutrients, such as protein, energy, B vitamins and calcium, are particularly important for the proper development of bones and teeth [162]. Several studies revealed that the exclusion of cow’s milk from the diet might lead to the impairment of bone health, short stature or weight deficit [151,163,164]. Infants with CMA were reported to have lower levels of vitamin D compared to healthy controls [165]. On the contrary, other authors failed to identify any significant difference regarding vitamin D status between CMA infants and healthy controls [166,167]. The contradictions become even more exacerbated, as a recent study performed on infants with CMA found no correlation between the serum level of vitamin D and eosinophilic cationic protein, an indicator of allergic diseases [161]. 

All these findings represent a burden for clinical practice in terms of correct workup of infants with CMA. Further studies including larger samples should be performed in order to provide an accurate evidence-based approach to these patients. 

### 2.6. Vitamin D and Diarrhea

Diarrhea represents one of the most common pathologies in children younger than the age of 5 years in developing countries and the second most common cause of morbidity and mortality related to infectious causes in these patients [168,169]. Given that vitamin D deficiency is related to susceptibility to and the severity of acute infections and poor outcomes in several chronic infections [170], it is not surprising that studies have been conducted with the aim of identifying the role of this vitamin in pediatric patients with diarrhea; however, the evidence remains scarce. Several studies from different geographic areas, such as Columbia, Egypt, Pakistan and Saudi Arabia, reported a significant association between low vitamin levels and increased incidence of diarrhea in children [171,172,173,174]. A recent study questioned these findings, proving that although children younger than five years were commonly found to be vitamin-D-deficient, this deficiency is not necessarily related to the incidence of diarrhea in this age group [175]. Similarly, Ahmed et al. found no association between vitamin D status and the incidence or severity of diarrhea in children aged 6–24 months [176]. Nevertheless, Wang et al. suggested that low vitamin D levels might be associated with ab increased likelihood of recurrence in patients with *Clostridium difficile*-associated diarrhea [177]. Similar findings were concluded in a study including patients with *Rotavirus* infections, with the authors proving that rotaviral diarrhea was associated with low vitamin D levels [178]. 

### 2.7. Vitamin D and Constipation

The relationship between vitamin D and chronic constipation is probably one of the less studied topics and; it remains unclear how vitamin D influences the motility of the gastrointestinal tract. It is well-documented that the presence of VDR on the surface of macrophages, lymphocytes and gut epithelial cells represents the key factor linking vitamin D deficiency, VDR dysfunction and altered gut microbial composition, eventually leading to the onset of several chronic conditions [179,180,181]. The relationship between vitamin D deficiency and both slow colonic motility and autonomic rectal dysfunction was previously proven in patients with multiple sclerosis [182,183,184,185]. Moreover, Panarese et al. recently hypothesized that vitamin D deficiency might be responsible for immunologic/metabolic damage to neuromuscular and epithelial components of the gut [186]. The authors proved that vitamin D levels were independently related to intestinal motility disorders. In addition, the symptoms of patients with functional chronic constipation were reported to worsen in parallel with decreased vitamin D levels. These findings remain to be further validated in larger studies focusing on the effect of vitamin D supplementation in patients with chronic functional constipation.

**Table 1:** The role of Vitamin D in pediatric digestive disorders.

Disease	Vitamin D Roles
**Gastritis and gastroesophageal reflux**	Increased eradication rates of *H. pylori* infection if vitamin D supplementation is combined with clarithromycin-based triple therapy [42] → protective antimicrobial effect against *H. pylori* infection Patients with *H. pylori*-positive gastritis: lower serum vitamin D concentrations compared to uninfected individuals [44] Vitamin D plays a major role in gastric mucosa homeostasis [45] Vitamin D receptor mRNA expression levels were significantly increased in patients with *H. pylori* infection and positively correlated with the activity scores of chronic inflammation [45]
**Celiac disease**	Vitamin D deficiency might be linked to the development of CD < 15 years of age [55] Negative effects of vitamin D deficiency might begin during intrauterine life, with maternal vitamin D deficiency implicated in the onset of CD [61,62] Positive effect of vitamin D on lymphocytes, as well as T and dendritic cells, which are involved in the regulation of gut barrier integrity, upregulating tight junction protein expression and consequently suppressing the increase in gut mucosa permeability [64,66] Vitamin D is involved in regulating inflammatory cytokines, such as TNF α, playing a crucial role in the maintenance of gut barrier integrity [68] Decreased expression of vitamin D receptor and epithelial barrier proteins claudin-2 and E-cadherin is positively correlated with histological findings of disease severity [69] A gluten-free diet alone lasting for approximately 6 months was sufficient to resolve hypocalcemia and hyperparathyroidism, as well as to normalize vitamin D levels [70] In teenagers with CD, a 2-year course of vitamin D (400 UI/day) and calcium (1 g/day) supplementation had a positive impact on bone mineral density [74]
**Cystic fibrosis**	Vitamin D deficiency (<10 ng/mL) in adults with advanced CF was positively correlated with reduced hip and spine bone mineral density compared to CF patients with higher vitamin D levels [88,89] In children, the concentration of vitamin D was positively correlated with femoral neck and lumbar spine Z scores [88,89] Vitamin D may be also involved in preserving lung function in patients with CF [90] In lung exacerbations and infections, locally produced 1,25(OH)2D enhances the IL-37 airway concentrations, further decreasing airway colonization by certain pathogens, such as *Pseudomonas aeruginosa* and *Bordetella bronchiseptica* [93] Vitamin D downregulates proinflammatory cytokine synthesis in macrophages, resulting in a reduction in CF airway inflammation [94] High-dose vitamin D supplementation decreases inflammatory biomarkers TNF α and IL-6 [96] High-dose vitamin D represents an adjunctive therapy in CF patients with acute airway infections [97] Vitamin D exerts anti-inflammatory effects in the intestine of CF patients, resulting in decreased synthesis of proinflammatory markers, reduced eosinophilia levels within the duodenal mucosa and fewer apoptotic cells [99] Vitamin D contributes to the development of healthier intestinal and respiratory microbes [101] Vitamin D exerted an anticatabolic effect in CF patients [102]
**Inflammatory bowel disease**	Vitamin D induces and maintains remission in IBD patients based on its anti-inflammatory and antibacterial properties [105,106] Vitamin D might also impact the incidence and progression of CD and UC [107,108,109] Vitamin D is involved in IBD-specific complications [112,113] Vitamin D levels might be higher in pediatric patients with UC compared to those with CD [114,115] Predictors of vitamin D deficiency in IBD include IBD-related surgery, non-white ethnicity [118,119], African-American ethnicity and body mass index > 30 kg/m^2^ [120] Serum vitamin D concentrations are inversely correlated with endoscopic and histologically proven inflammation, mucosal expression of proinflammatory cytokines and disease activity [128,129] Low or insufficient vitamin D levels are related to increased need for hospitalization and surgery in patients with IBD compared to those with normal serum levels [131,132] Promising effects of vitamin D on IBD gut microbiota modulation [9] Supplementation of vitamin D in IBD is associated with an improvement in inflammatory biomarkers, suppression of Th1 immune response and reduced clinical disease activity index [136,137,138,139,140,141,142,143]
**Food allergy**	Vitamin D insufficiency supports the development of food allergies [144] Vitamin D receptor agonists influence Th1 and Th2 cell function, inhibiting the maturation of dendritic cells and inducing tolerogenic dendritic cells, as well as contributing to the induction of regulatory CD4^+^CD25^+^Foxp3^+^ T cells [145] Vitamin D deficiency increases gut permeability due to colonization by pathogenic microbial flora, resulting in inadequate immune system exposure to dietary allergens [146], allergic sensitization, eczema and food allergies [147] Vitamin D deficiency is associated with the development of atopic dermatitis, asthma and food allergies [152] IgE- and non-IgE-mediated food allergies are associated with increased risk of vitamin D deficiency [155,156,157] Higher levels of vitamin D might increase the likelihood of sensitization and food allergies [158] The correction of vitamin D deficiency has a positive impact on food allergy prognosis, with a domino effect between inadequate sunlight exposure, vitamin D deficiency, altered gut barrier integrity, impaired immune response and food allergies [160] Infants with CMA present with lower levels of vitamin D compared to healthy controls [165] No correlation between the serum level of vitamin D and eosinophilic cationic protein, which is an indicator of allergic diseases [161]
**Diarrhea**	Significant association between low vitamin levels and the increased incidence of diarrhea in children [171,172,173,174] No association between vitamin D status and incidence or severity of diarrhea in children aged 6–24 months [176] Low vitamin D levels associated with increased likelihood of recurrence in patients with *Clostridium difficile*-associated diarrhea [177]
**Constipation**	Vitamin D deficiency might be responsible for immunologic/metabolic damage to neuromuscular and epithelial components of the gut [186] Vitamin D levels are related to intestinal motility disorders [186] The symptoms of patients with functional chronic constipation worsened in association with a decrease in vitamin D levels [186]

## 3. Conclusions

Vitamin D plays a crucial role in terms of gastrointestinal health. Thus, besides the classical role of vitamin D in calcium metabolism and bone health, emerging evidence indicates a wide spectrum of other multisystemic implications. The antimicrobial effect is extremely useful in enhancing *H. pylori* eradication. Studies of pediatric patients proved that supplementation of vitamin D in combination with standard eradication regimens increases *H. pylori* eradication rates. In terms of gastroesophageal reflux, the evidence in pediatric ages remains scarce, suggested that these children have normal levels of vitamin D despite their low intake. Moreover, this beneficial micronutrient acts as an immunomodulator of both innate and adaptive immune responses at the level of the gastrointestinal tract. In terms of CD, vitamin D deficiency is involved not only in the onset of CD, but it might also aggravate its clinical course. Therefore, early disruption of the intestinal barrier in susceptible individuals and subsequently increased permeability triggered by vitamin D deficiency could contribute to the onset of immune responses triggered by gluten. Maternal vitamin D status might also be involved in the early onset of CD in genetically susceptible offspring. The impact of vitamin D on CF is complex, as it was proven to exert antimicrobial, modulatory and anticatabolic effects. Moreover, vitamin D deficiency in CF children was proven to be positively correlated with reduced bone mineral density. Vitamin D may be also involved in preserving lung function in patients with CF. The supplementation of vitamin D in CF patients might enhance the development of healthier intestinal and respiratory microbes, improving the composition of the lung and gut microbiome. The role of vitamin D on maintaining gut barrier integrity was demonstrated to be of considerable importance in IBD patients. Moreover, based on its immunomodulatory properties, this vitamin seems to decrease disease activity in these patients, preventing IBD-related complications. Pediatric patients with ChD were proven to have lower vitamin D levels compared to those with UC. Children with food allergies were reported to have lower levels of vitamin D, and vitamin D was found to increase the risk of developing food allergies in the pediatric population. CMA infants were found to have lower levels of vitamin D levels compared to controls, although the reported findings remain controversial. Several studies worldwide have reported that low vitamin D levels are associated with increased incidence of diarrhea in children; however, other authors found no association between vitamin D status and increased likelihood of diarrhea. Vitamin D was recently linked to the development of intestinal motility dysfunctions, proving that vitamin D deficiency might increase the risk of chronic constipation, worsening its associated symptoms. Nevertheless, further studies including larger samples should be performed in order to define the precise systemic role of vitamin D and to elucidate the related ‘chicken or egg’ controversies.

## Data Availability

Not applicable.

## Abbreviations

CD	celiac disease
CF	cystic fibrosis
CFTR	cystic fibrosis conductance regulator
ChD	Crohn’s disease
CMA	cow’s milk allergy
*H. pylori*	*Helicobacter Pylori*
IBD	inflammatory bowel disease
IL	interleukin
RXR	retinoic acid receptor
UC	ulcerative colitis
VDR	vitamin D receptor
VDRE	vitamin D response element
